# Difference in the neurocognitive functions of WLWH and MLWH in an Italian cohort of people living with HIV

**DOI:** 10.1007/s13365-022-01078-z

**Published:** 2022-06-19

**Authors:** Valentina Delle Donne, Valentina Massaroni, Nicoletta Ciccarelli, Francesca Lombardi, Alberto Borghetti, Arturo Ciccullo, Alex Dusina, Damiano Farinacci, Ganmaria Baldin, Elena Visconti, Enrica Tamburrini, Simona Di Giambenedetto

**Affiliations:** 1 Department of Safety and Bioethics, Infectious Diseases Institute, Catholic University of Sacred Heart, Largo Francesco Vito 1; 00168, Rome, Italy; 2 Department of Psychology, Catholic University, Milan, Italy; 3 UOC Infectious Diseases, Fondazione Policlinico Universitario A. Gemelli IRCCS, Rome, Italy; 4 UOC Infectious Diseases, Ospedale S. Salvatore, L’Aquila, Italy; 5 UOC Infectious Diseases, Mater Olbia Hospital, Olbia, Italy

**Keywords:** HIV, HIV-associated neurocognitive disorders, Sex differences, Neurocognition, Cognition evaluation

## Abstract

Based on the available literature, women living with HIV (WLWH) seem to show greater cognitive and emotional disadvantages than men living with HIV (MLWH). Our aim was to compare the cognitive performance of MLWH and WLWH in an Italian cohort of People Living With HIV (PLWH) and to analyse factors potentially contributing to sex differences in cognitive function. We ran a retrospective, cross-sectional analysis of a monocentric dataset of PLWH who were administered a standardized neuropsychological test battery (SNB) during routine clinical care. We enrolled 161 Italian PLWH who are on combined antiretroviral therapy (cART): 114 (70.8%) MLWH and 47 (29.2%) WLWH.

Global cognitive performance (composite *z* score) (GCP) was significantly higher in MLWH than WLWH [mean 0.19 (SD 0.85) vs − 0.13 (SD 0.96); *p* = 0.039]. Moreover, WLWH obtained significantly higher scores on the Zung Depression Scale than MLWH [mean 41.8 (SD 10.9) vs 36.7 (SD 9.2); *p* = 0.003]. However, there was no statistically significant direct effect between male sex and better GCP (*p* = 0.692) in the context of a mediation model. On the contrary, the associations between male sex and better GCP were mediated by higher level of education (a*b =  + 0.15, Bootstrap CI95 = 0.05 and 0.27) and a lower Zung depression score (a*b =  + 0.10, Bootstrap CI95 = 0.02 and 0.21).

In conclusion, the global cognitive performance of WLWH is lower than that of MLWH. However, other demographic and clinical factors besides sex might help explain differences in their neurocognitive functions and make it possible for us to monitor them and identify those patients most in need.

## Introduction

There are several issues relating to the HIV care that make it important to differentiate women living with HIV (WLWH) from men living with HIV (MLWH) (Geretti et al. 2017). Indeed, sex-specific factors may impact on behaviours and conditions of PLWH, affecting access to diagnosis and treatment, clinical management, treatment response, and the overall wellbeing (Rosin et al. 2015; Lundgren et al. 2015; Soon et al. 2012; Burch et al. 2016; Ribaudo et al. 2016). For instance, WLWH demonstrated lower adherence to antiretroviral therapy (Puskas et al. 2011) and, despite a lower viral load, they did not show appreciable benefits in terms of disease progression compared to MLWH (Perez-Elias et al. 2014; Law et al. 2015; Napravnik et al. 2002). Regarding studies on HIV and cognition, neurocognitive studies focusing on WLWH are increased since the introduction of effective combined antiretroviral therapy (cART) (Maki et al. 2018; Sundermann et al. 2018; Rubin et al. 2020).

Available data suggest that WLWH have some cognitive and emotional disadvantages compared to MLWH. A recent systematic American review assessed whether WLWH are more cognitively vulnerable than MLWH (Rubin et al. 2019) and concluded that few HIV studies are adequately powered to address sex differences in prevalence and pattern of HAND. However, several studies indicate that WLWH have a higher prevalence of HAND (Sundermann et al. 2018; Royal et al. 2016) and a lower performance or higher impairment of memory and learning (Kabuba et al. 2016; Royal et al. 2016), speed of information processing (Maki et al. 2018; Do et al. 2018) and motor abilities (Maki et al. 2018; Burlacu et al. 2018) than MLWH. Only one study reported lower performance of WLWH in executive functions (Maki et al. 2018), and no difference was observed for fluency or attention/working memory (Do et al. 2018; Sundermann et al. 2018; Kabuba et al. 2016; Burlacu et al. 2018; Royal et al. 2016; Behrman-Lay et al. 2016).

Several factors seem to contribute to documented sex differences. Among biological factors (e.g., inflammation, hormones, genetics) (Rubin et al. 2019), there is evidence of an association between testosterone insufficiency and cognitive complaints (Laan et al. 2019) and of sex differences in inflammatory biomarkers (Ticona et al. 2015; Fitch et al. 2013; Martin et al. 2013; Looby et al. 2016). The higher prevalence of HAND in WLWH compared to MLWH could also be linked to differences in other variables, such as socio-economic conditions and education (Basso and Bornstein 2000; Farinpour et al. 2003). These factors could be some of the cognitive reserve determinants prior to HIV infection and might contribute to greater cognitive vulnerability after infection (Tsai and Burns 2015; Singer 1994).

In fact, in a recent study, Rubin et al. (2020) suggested that despite different cognitive profiles according to sex, the most discriminative factors were similar between men and women and included reading level (cognitive reserve), current and nadir CD4 count, plasma HIV viral load, duration of HIV disease, age, depressive symptoms, and race/ethnicity.

Furthermore, mental health disorders might actually contribute to higher rates of HAND in WLWH versus MLWH. Indeed, in a recent review, Geretti et al. (2017) suggested that WLWH experience a disproportionate burden of mental health issues (Orza et al. 2015) and face very different emotional challenges upon diagnosis (Loutfy et al. 2013). Specifically, the higher frequency of cognitive impairment in WLWH compared to MLWH might be related to the higher prevalence of depression (Bayón et al. 2012; Morrison et al. 2002; Rabkin et al. 1991; Semple et al. 1996; Aljassem et al. 2016). In fact, depression is negatively associated with cognitive functioning in PLWH and both a history of depression and current depression are associated with HAND in PLWH (Yousuf et al. 2019). Several international studies suggest that depression is almost twice as common in women as in men (Yousuf et al. 2019; Fantahun et al. 2018; Antelman et al. 2007) and that WLWH may be more cognitively susceptible than MLWH to the effects of mental health factors (Rubin et al. 2019, 2020). In particular, Rubin et al. (2020) indicated that HIV comorbid with depression affects specific cognitive domains, including cognitive control, and that these effects are largest in women. Other studies reported that depression is associated with deficits in learning, memory, and attention in WLWH (Maki et al. 2018; Rubin et al. 2016, 2014, 2015) and that women with elevated depressive symptoms seem to have a three times greater risk of impairment in executive control/inhibition than MLWH with elevated depressive symptoms (Rubin et al. 2019).

However, to date, few studies have been published on this topic, especially in Italy where literature recounts inconsistent results in PLWH. For example, Focà et al. (2016) reported, in contrast with other previous studies, that MLWH might be most at risk for neurocognitive disorders than WLWH.

Thus, as significant knowledge gaps remain, additional studies are needed to deepen this subject and improve management of PLWH.

Our aim was to compare the cognitive performance of MLWH and WLWH in an Italian Cohort of People Living With HIV and to analyse factors potentially contributing to sex differences in cognitive function.

## Methods

### Participants

We performed a retrospective, cross-sectional analysis of a monocentric dataset that included PLWH who are being followed at the Infectious Diseases Institute of “Policlinico Gemelli Foundation” of Rome, and who were submitted to a standardized neuropsychological test battery (SNB) during routine clinical care between January 2011 and March 2019.

Data concerning the following demographic, clinical, and laboratory variables were collected for each participant at the time of the neuropsychological testing: age, education, ethnicity, risk factors for HIV infection, time from HIV diagnosis, current and past antiretroviral regimen, current and nadir CD4 cell count, HIV-1 viral load, co-infection with hepatitis C virus (HCV), comorbidities, use of antidepressant and concomitant medications.

### Procedure

#### Neuropsychological examination

By means of SNB we investigated the following cognitive areas:

- learning memory: The Rey Auditory Verbal Learning Test – RAVLT;
- attention: WAIS Digit Span, WAIS Digit Symbol;
- verbal fluency: FAS Test (subtest of the Neurosensory Center Comprehensive Examination for Aphasia-NCCEA);
- executive functioning: Multiple features target cancellation- MFTC;
- fine motor skills: Grooved Pegboard Test.

We measured global cognitive performance (GCP) by transforming raw scores obtained on each task into standardized *Z* scores using means and standard deviations of Italian normative data (Capitani and Laiacona 1997; Carlesimo et al. 1996) and averaging them to calculate a composite total score and a score for each domain score. Domain-specific impairment was defined as a *Z* score less than −1. According to the Frascati Criteria (Antinori et al. 2007), patients were considered affected by HIV-associated neurocognitive diseases (HAND) if they showed impairment in at least two domains.

Each patient was also administered the Zung Self-Rating Depression Scale (Zung 1965, 1986), a short self-report questionnaire that investigates four common characteristics of depression: the pervasive effect, the physiological equivalents, other disturbances, and psychomotor activities. A score ≥ 50 is considered to indicate probable depression and abnormal/elevated depression symptoms.

### Statistical analysis

Descriptive statistics were calculated for quantitative variables [median, interquartile range (IQR), means, standard deviations (SD)] and qualitative variables (percent frequencies).

When appropriate, *T* tests or the Chi-square test were performed to determine differences in cognitive performance and Zung scores among groups based on sex: WLWH = group 1 and MLWH = group 2.

We also computed Cohen’s *d* in order to identify effect sizes and show the clinical significance of comparisons between groups 1 and 2.

We performed a mediation analysis through a bootstrapping method using SPSS Process Macro to examine the effect of mediating variables on the relationship between sex and cognitive performance (Abu-Bader and Jones 2021).

We selected the most discriminating factors based on the literature (Basso and Bornstein 2000; Farinpour et al. 2003; Rubin et al. 2020). Thus, we examined as mediators the following collected variables: age, education, injecting drug use, time from HIV diagnosis, time from cART, current and nadir CD4 cell count, HIV-1 viral load, past AIDS-defining events and co-infection with HCV.

According to Baron and Kenny (1986), we chose demographic, clinical, and laboratory variables as mediators when regression analyses revealed three statistically significant relationships: (1) sex was a statistically significant predictor of global or domain-specific cognitive performance. (2) Sex was a statistically significant predictor of the mediator. (3) The mediator was a statistically significant predictor of the global or domain-specific cognitive performance (dependent variable) while controlling for the effect of sex.

All analyses were performed using the SPSS version 21.0 software package (SPSS Inc., Chicago, IL).

## Results

### Demographic and clinical characteristics

A total of 161 Italian PLWH on cART were enrolled: 114 (70.8%) MLWH and 47 (29.2%) WLWH. Their demographic and clinical characteristics are summarized in Table 1.

**Table 1:** Demographic and clinical characteristics of WLWH (*n* = 47) and MLWH (*n* = 114)

**Parameters**	**WLWH** *N* (%) o median (IQR)*	**MLWH** *N* (%) o median (IQR)*	***p***
Age, years*	50 (44–56)	51 (42–57)	0.096
Education, years*	13 (8–13)	13 (10–17)	**0.006**
Time from HIV diagnosis, years*	17 (11–22)	10 (4–19)	**0.020**
Time from cART, years*	14 (6–17)	8 (3–15)	**0.009**
HIV-RNA < 50 copie/ml	38 (81)	102 (89.5)	0.383
CD4 cell count, cell/µL*	637 (448–895)	569 (389–723)	0.062
CD4 nadir cell count, cell/µL*	171 (65–249)	150 (65–284)	0.094
Injecting drug users	10 (21.3)	12 (10.5)	0.057
Past AIDS-defining events	12 (25.5)	32 (28)	0.742
Hepatitis coinfection	10 (21.3)	18 (15.8)	0.310
Use of antidepressants	7 (15)	10 (8.8)	0.250

Bold values represent statistically significant *p* values

*N* number, *IQR* interquartile range, *WLWH* women living with HIV, *MLWH* men living with HIV, *cART* combined antiretroviral therapy

^*^Median and IQR reported

The subjects in the two groups (MLWH vs WLWH) significantly differed in mean education [mean 13 (SD 3.6) vs 11 (SD 3.1); *p* = 0.006], with a medium effect size (*d* = 0.59), time from HIV diagnosis [mean 12.7 (SD 9.3) vs 16.6 (SD 8.7); *p* = 0.020], with a small effect size (*d* = 0.43), and from first cART [mean 9.9 (SD 7.4) vs 12.8 (SD 7.5); *p* = 0.009], with a small effect size (*d* = 0.38).

### Neuropsychological performance in MLWH and WLWH

GCP was significantly higher in MLWH than WLWH [mean 0.19 (SD 0.85) vs −0.13 (SD 0.96); *p* = 0.039] with a small effect size (*d* = 0.35). Considering each cognitive domain separately, MLWH performed better than WLWH on attention [mean 0.16 (SD 0.73) vs −0.26 (SD 0.69); *p* < 0.001], with a medium effect size (*d* = 0.59), and executive functions [mean 0.02 (SD 0.80) vs −0.26 (SD 0.78); *p* = 0.002], with a small effect size (*d* = 0.35). Nevertheless, the percentage of patients affected by HAND did not significantly differ between MLWH and WLWH [20/114 (17.5%) vs 13/47 (27.7%), *p* = 0.148].

Moreover, WLWH obtained significantly higher scores on the Zung Depression Scale than MLWH [mean 41.8 (SD 10.9) vs 36.7 (SD 9.2); *p* = 0.003], with a medium effect size (*d* = 0.50), indicating more symptoms of depression in the former group and a significantly higher percentage of subjects with probable depression according to the Zung Depression normative cut-off [23.4% (*n* = 11/47) vs 8.8% (*n* = 10/114); *p* = 0.012]. Results of the neuropsychological performances in each cognitive domain are shown in Table 2.

**Table 2:** Neuropsychological performances in each cognitive domain

**Cognitive domains**	**WLWH**		**MLWH**	***N* (%) of patients** **with domain-specific impairment**^**a**^	***p***^**c**^
	**Mean (SD)**	***N* (%) of patients** **with domain-specific impairment**^**a**^	**Mean (SD)**		
Global *Z* score^b^	−0.13 (0.96)	5 (10.6)	0.19 (0.85)	8 (7)	**0.039**
Memory	−0.30 (1.21)	14 (29.8)	−0.41 (1.13)	27 (23.7)	0.422
Attention	−0.26 (0.69)	7 (14.9)	0.16 (0.73)	10 (8.8)	**< 0.001**
Executive functions	−0.26 (0.78)	6 (12.8)	0.02 (0.80)	14 (12.3)	**0.002**
Language	0.53 (1.25)	6 (12.8)	0.60 (1.12)	10 (8.8)	0.711
Fine motor skills	−0.39 (2.22)	8 (17)	0.29 (2.03)	18 (15.8)	0.065
Zung Depression Scale	41.8 (10.9)	11 (23.4)	36.7 (9.2)	10 (8.8)	**0.003**

Bold values represent statistically significant *p* values

*N* number, *SD* standard deviation, *WLWH* women living with HIV, *MLWH* men living with HIV

^a^Based on Italian normative data

^b^Average of single *Z* score on each domain

^c^*p* values related to the *T* test that compared the means

### Factors contributing to sex differences in cognitive function

We used linear regression analysis to select demographic, clinical, and laboratory variables as mediators intervened between sex and cognitive performance. First, the results of the regression analysis show that male sex was a significant predictor of higher level of education (mean change + 1.76; 95% CI 0.56/2.96; *p* = 0.004) and of a lower Zung depression score (mean change − 0.51; 95% CI −8.44/ −1.78; *p* = 0.003).

Next, while controlling for sex, the results of the second regression analysis show that higher level of education was a significant predictor of better GCP (mean change + 0.09 per 1 year increase; 95% CI 0.05/0.13; *p* < 0.001) and attention skills (mean change + 0.07 per 1 year increase; 95% CI 0.05/0.10; *p* < 0.001). Furthermore, after controlling for sex, a lower Zung depression score was a significant predictor of better GCP (mean change −0.02 per 1 score; 95% CI − 0.03/ −0.01; *p* = 0.001), attention skills (mean change −0.02 per 1 score; 95% CI −0.03/ −0.01; *p* < 0.001) and executive skills (mean change −0.01 per 1 score; 95% CI −0.02/ −0.03; *p* = 0.019).

We performed a bootstrapping method to examine if education and Zung depression score mediated the relationship between sex and GCP (Table 3), attention and executive skills.

**Table 3:** Mediation analysis to examine direct and indirect effects of sex on GCP

Effects of sex on GCP and mediators*	***b***	***SE***	***t***	***p***	**95% CI**
Sex (male vs female) → GCP	0.32	0.15	2.08	**0.039**	0.01/0.62
Sex (male vs female) → education (per 1-year increase)	1.76	0.6	2.89	**0.004**	−8.44/−1.78
Sex (male vs female) → Zung Depression Scale (per 1 point increase)	−5.11	1.58	−3.03	**0.028**	−8.44/1.78
Sex (male vs female) → education (per 1-year increase) → GCP	0.15	0.01	5.07	**< 0.001**	0.05/0.13
Sex (male vs female) → Zung Depression Scale (per 1 point increase) → GCP	0.02	0.007	−3.52	**0.001**	−0.03/ −0.01
Direct and indirect effects of sex on GCP**					
Direct (c)	0.05	0.14	0.39	0.692	−0.23/0.34
Indirect^a^ (a*b)					
Zung Depression Scale	0.10	0.04			
Education	0.15	0.05			
Total (*c* + a*b)	0.32	0.15	2.08	**0.038**	0.01/0.62

Bold values represent statistically significant *p* values

*CI* confidence interval, *SE* standard error, *t* test statistic, *GCP* Global cognitive performance

^*^Education and Zung Depression Scale are the only two mediators in the light of the method that provides these conditions: “(1) sex was a statistically significant predictor of global cognitive performance. (2) Sex was a statistically significant predictor of the mediator. (3) The mediator was a statistically significant predictor of the global cognitive performance while controlling for the effect of sex”; ^**^Direct effect examines if the relationship between sex and GCP is direct and not mediated by a third variable; indirect effect examines the null hypothesis that the indirect relationship between the sex and GCP is equal to zero; total effect, effect produced by the entire model, indirect and direct effect

^a^Based on 5000 bootstrap samples

The results of the indirect effect based on 5000 bootstrap samples showed the following associations. It emerged a significant indirect relationship (a*b) between male sex and better GCP and attention skills mediated by higher level of education (a*b =  + 0.15, Bootstrap CI95 = 0.05 and 0.27; a*b =  + 0.12, Bootstrap CI95 = 0.04 and 0.22, respectively) and a lower Zung depression score (a*b =  + 0.10, Bootstrap CI95 = 0.02 and 0.21; a*b =  + 0.10, Bootstrap CI95 = 0.02 and 0.20, respectively). Furthermore, it emerged a significant indirect relationship (a*b) between male sex and better executive skills mediated by a lower Zung depression score (a*b =  + 0.07, Bootstrap CI95 = 0.00 and 0.16).

The mediator, education, accounted for approximately 46% of the total effect on GCP [*P*_M_ = (0.15) / (0.32)] and for approximately 27% of the total effect on attention skills [*P*_M_ = (0.12) / (0.43)]. Moreover, the mediator, Zung depression score, accounted for approximately 32% of the total effect on GCP [*P*_M_ = (0.10) / (0.32)], for approximately 23% of the total effect on attention skills [*P*_M_ = (0.10) / (0.43)] and for approximately 24% of the total effect on executive skills [*P*_M_ = (0.07) / (0.29)].

On the other hand, there was no statistically significant direct effect (*c*) between sex and GCP (*b* =  + 0.57, *t* = 0.39, *p* = 0.692), attention skills (*b* =  + 0.20, t = 1.72, *p* = 0.087), and executive skills (*b* =  + 0.21, t = 1.72, *p* = 0.125) in the context of the mediation model.

## Discussion

The aim of this study was to analyse the cognitive profile of MLWH and WLWH in an Italian cohort of PLWH and to examine the effect of mediating variables on the relationship between sex and cognitive function. Indeed, based on the available literature, WLWH seem to have cognitive and emotional disadvantages (Rubin et al. 2019; Bayón et al. 2012; Morrison et al. 2002). However, a significant knowledge gap remains on this topic especially in Italy where few studies have been published with inconsistent results (Focà et al. 2016).

Overall, the WLWH in our cohort of patients had a lower level of education and longer time from HIV diagnosis and from first cART compared to MWLH. In line with previous evidence (Sundermann et al. 2018; Royal et al. 2016), our findings show that WLWH demonstrate lower global cognitive performance than MLWH and a higher proportion of subjects with probable depression. However, our WLWH did not manifest a significantly higher frequency of HAND compared to MLWH, in agreement with the Frascati criteria (Antinori et al. 2007). This discrepancy could be explained by the fact that WLWH demonstrate mild impairment than MLWH across domains that does not typically meet the threshold of impairment contributing to a HAND diagnosis.

In this regard, discrepant data emerged from previous observations. For example, Sundermann et al. (2018) found a higher prevalence of HAND in WLWH compared to MLWH; however, this difference was not confirmed by adjusting for reading level and ethnicity. On the other hand, from Rubin et al.’s review (2019), it emerged that only 43% of the studies examined demonstrated that the prevalence of HAND was higher in WLWH. Considering cognitive domains individually, we found additional differences. First, WLWH performed worse than MLWH in the executive skills domain, confirming previous observations (Maki et al. 2018). Furthermore, our WLWH sample also performed worse than MLWH on the attention tasks; note, however, that in this domain, there are inconsistent results across studies (Do et al. 2018; Sundermann et al. 2018; Kabuba et al. 2016; Burlacu et al. 2018; Royal et al. 2016; Behrman-Lay et al. 2016) probably due to methodological differences.

However, we identified other demographic and clinical factors besides sex that could help explain the aforementioned difference between WLWH and MLWH. Indeed, our results showed that there was no statistically significant direct effect between sex and GCP, attention and executive skills. On the contrary, the associations between male sex and better GCP and attention skills were mediated by higher level of education and fewer depressive symptoms. Moreover, a lower Zung depression score mediated the association between male sex and better executive skills.

Thus, as suggested in previous studies, we confirm that depressive symptoms seem to be more prevalent in WLWH than in MLWH (Aljassem et al. 2016) and might contribute to higher cognitive impairment (Bayón et al. 2012). Furthermore, we confirm several data reported in the literature regarding predictive factors for cognitive performance in PLWH such as educational background (Tedaldi et al. 2015; Milanini et al. 2016).

Indeed, we corroborate the finding that adjusting for critical factors such as education, clinical variables, and mental health reduces the dimension of the sex difference and elucidates factors contributing to NCI in women (Rubin et al. 2019). Moreover, our findings support those of Sundermann et al. (2018), suggesting that discrepancies in biopsychosocial factors (e.g., reading level that reflect education quality) may explain the sex difference in NCI regarding PLWH.

We acknowledge that our study has some limitations. First, our conclusions are limited by the small sample size of the study. Indeed, due to the small number of patients who were submitted to a completed SNB in the dataset used for the retrospective analysis, our study reached a low statistical power. Thus, in future studies, it will be important to extend the sample to confirm the generalizability of the results achieved.

Second, this is a cross-sectional study and uncontrolled biases can occur in routine clinical practice. Thus, future longitudinal studies are needed to confirm our findings also given the fact that only one longitudinal study to date has examined the risk of neurocognitive decline in WLWH and MLWH (Heaton et al. 2015).

Lastly, in future investigations, it would be useful to include a healthy control group to compare differences in cognitive functions between women and men and between PLWH and the general population. In addition, our WLWH and MLWH subgroups were not matched for education, years from HIV diagnosis and years from first ARV; future investigations that include matched groups are needed to check the probable influences of these disparities on cognitive performance.

In conclusion, taken together, our findings suggest that in our sample, WLWH demonstrate lower global cognitive performance, attention, and executive skills compared to MLWH. However, other demographic and clinical factors might be able to explain these disadvantages in WLWH besides sex, in particular the lower level of education and more depressive symptoms. On these bases, in planning cognitive interventions, it seems appropriate to pay particular attention to WLWH, but it would also be helpful to monitor demographic, clinical, and mental health factors to identify the patients who are most in need and to detect cognitive impairments as soon as possible and intervene to improve the psychological wellbeing of PLWH.
